# Predictable ecological response to rising CO_2_ of a community of marine phytoplankton

**DOI:** 10.1002/ece3.3971

**Published:** 2018-04-02

**Authors:** Jacob Pardew, Macarena Blanco Pimentel, Etienne Low‐Decarie

**Affiliations:** ^1^ University of Essex Colchester UK

**Keywords:** competition coefficient, global change, primary producers, taxonomic group

## Abstract

Rising atmospheric CO_2_ and ocean acidification are fundamentally altering conditions for life of all marine organisms, including phytoplankton. Differences in CO_2_ related physiology between major phytoplankton taxa lead to differences in their ability to take up and utilize CO_2_. These differences may cause predictable shifts in the composition of marine phytoplankton communities in response to rising atmospheric CO_2_. We report an experiment in which seven species of marine phytoplankton, belonging to four major taxonomic groups (cyanobacteria, chlorophytes, diatoms, and coccolithophores), were grown at both ambient (500 μatm) and future (1,000 μatm) CO_2_ levels. These phytoplankton were grown as individual species, as cultures of pairs of species and as a community assemblage of all seven species in two culture regimes (high‐nitrogen batch cultures and lower‐nitrogen semicontinuous cultures, although not under nitrogen limitation). All phytoplankton species tested in this study increased their growth rates under elevated CO_2_ independent of the culture regime. We also find that, despite species‐specific variation in growth response to high CO_2_, the identity of major taxonomic groups provides a good prediction of changes in population growth and competitive ability under high CO_2_. The CO_2_‐induced growth response is a good predictor of CO_2_‐induced changes in competition (*R* ^2^ > .93) and community composition (*R* ^2^ > .73). This study suggests that it may be possible to infer how marine phytoplankton communities respond to rising CO_2_ levels from the knowledge of the physiology of major taxonomic groups, but that these predictions may require further characterization of these traits across a diversity of growth conditions. These findings must be validated in the context of limitation by other nutrients. Also, in natural communities of phytoplankton, numerous other factors that may all respond to changes in CO2, including nitrogen fixation, grazing, and variation in the limiting resource will likely complicate this prediction.

## 1 INTRODUCTION

Atmospheric CO_2_ concentrations recently exceeded a historic high of 400 parts per million (μatm), a level which has not been surpassed in at least the past 420,000 years (Griggs & Noguer, 2002). This increase in atmospheric CO_2_ has led to a decrease in the ocean pH of 0.1 units since the onset of the Industrial Revolution (IPCC, 2014). The concentration of CO_2_ in the atmosphere is expected to continue to increase at a rate which is ten times faster than has been recorded in the past 55 million years (Almén et al., 2016). Predictions suggest that by the end of this century, atmospheric CO_2_ levels may reach between 700 and 1,000 μatm and ocean pH will decrease to ~7.8–7.7 (Brewer et al., 2014). These changes in atmospheric CO_2_ concentration and in ocean pH combine to increase the availability of CO_2_ for marine primary producers, hamper the ability of organisms to calcify and affect the growth rates of marine phytoplankton (Kroeker, Kordas, Crim, & Singh, 2010; Reinfelder, 2011).

Major phytoplankton taxonomic groups differ in the efficiency of their RuBisCO (Ribulose‐1,5‐bisphosphate carboxylase/oxygenase) activity and of their carbon concentration mechanisms (CCMs) and thus in their expected response to the rise in CO_2_ and ocean acidification (Reinfelder, 2011; Tortell, 2000). Those phytoplankton that are more efficient at taking up and utilizing carbon (higher surface area to volume ratio, higher CCM efficiency, higher RuBisCO specificity, and/or lower carbon requirements) are hypothesized to benefit less from the increase in CO_2_ as their growth is currently least CO_2_‐limited (Reinfelder, 2011). Cyanobacteria possess a highly specialized CCM involving carboxysomes to deal with low dissolved CO_2_ levels and a RuBisCO with CO_2_ specificity comparable to that of chlorophytes (Price, Badger, Woodger, & Long, 2008; Tortell, 2000). Cyanobacteria are capable of increasing the concentration of CO_2_ at the site of photosynthesis over 1,000 times the levels of its surrounding environment (Badger & Price, 2003), which is an order of magnitude higher carbon concentration efficiency than the next most efficient major taxonomic groups (Tortell et al., 2008). Although cyanobacteria are capable of increasing their growth rate under elevated CO_2_ [e.g., two species test in Fu, Warner, Zhang, Feng, and Hutchins (2007)], the magnitude of this increase in growth is generally considered to be small, and thus cyanobacteria would be expected to decrease in competitive ability under elevated CO_2_ all else being equal. However, nitrogen‐fixing cyanobacteria (diazotrophic cyanobacteria) are found to increase nitrogen fixation under elevated CO_2_ and have a greater increase in growth rate than other groups of phytoplankton (Dutkiewicz et al., 2015). Diazotrophic cyanobacteria likely differ from other cyanobacteria in their carbon uptake and utilization, however, part of their larger response and thus part of this difference between diazotrophs and nonnitrogen‐fixing cyanobacteria, may be attributed to culture conditions, in which nitrogen is completely omitted when culturing dizaotrophes for the measurement of nitrogen fixation but other nutrients are supplied in abundance (e.g., Hutchins, Fu, Webb, Walworth, & Tagliabue, 2013). Similarly to expectations for cyanobacteria that do not fix nitrogen, some diatoms have been shown to exhibit a limitation response only at CO_2_ concentrations below present‐day levels, suggesting that they also have a highly efficient CCM (Hopkinson, Dupont, Allen, & Morel, 2011), however, other diatoms do appear to have a large increase in growth under future CO_2_ conditions (e.g., Wu et al.*,* 2010). Chlorophytes and coccolithophores have a lower RuBisCO specificity than diatoms and lower CCM efficiency than dinoflagellates and cyanobacteria but comparable efficiency to that of diatoms (Price et al., 2008; Tortell, 2000). Chlorophytes and coccolithophores are therefore expected to be most limited by modern day CO_2_ availability and would thus be expected to benefit most from the increase in CO_2_ and increase in relative abundance. However, the benefits of higher CO_2_ for the photosynthesis of coccolithophores may be offset by its effect on calcification. Calcification is made costlier by the decrease in pH associated with rising atmospheric CO_2_ levels. Calcification in coccolithophores is generally hampered by the extent of acidification expected in this century, but the effects appear to be species‐specific (Meyer & Riebesell, 2015), so that it is not clear that coccolithophores will have a consistent change in growth and abundance under elevated CO_2_. Although there are substantive variation and uncertainty within major taxonomic groups for these traits, given the major taxa or species‐specific differences in carbon uptake and utilization and the differences between calcifying and noncalcifying phytoplankton species in response to acidification, it may be possible to predict some of the shifts in community assemblages under increasing CO_2_, with an expected decrease in nondiazotrophic cyanobacteria, an increase in chlorophytes and an intermediate response of diatoms.

A model parametrized using a meta‐analysis of growth response to increased CO_2_ and assuming that this growth response will directly translate into proportional changes in community composition has led to the expectation that major changes in the composition of communities should be expected with rising CO_2_ and that these changes will exceed those caused by warming or changes in nutrient availability (Dutkiewicz et al., 2015). The assumption that growth response will directly translate into proportional changes in community composition remains to be tested. Despite these important differences in CO_2_‐related physiology between major taxonomic groups, few studies have focused on how these differences will translate into changes in competition between groups and, ultimately, how they alter community composition with increasing CO_2_.

Studies of natural assemblages of marine phytoplankton have found that high CO_2_ resulted in an increase in the abundance of the cyanobacterium *Synechococcus* and a decrease in the abundance of the coccolithophore *Emiliania huxleyi* (Paulino, Egge, & Larsen, 2007). Similar experiments also found a decrease in fucoxanthin‐containing phytoplankton including diatoms (Yoshimura et al., 2009) or that community composition remains unchanged (Bermúdez et al., 2016). Within taxonomic groups, an increase in CO_2_ benefits larger over smaller diatoms (Tortell et al., 2008) as expected from facilitation of CO_2_ diffusion with larger surface area to volume ratios. Changes in composition of marine phytoplankton communities associated with changes in CO_2_ concentration could potentially be predicted from published information on the CO_2_‐related physiology associated with major taxonomic groups (including CCM efficiency and RuBisCO specificity) or the measurement of growth response to CO_2_ of the phytoplankton species present in the natural assemblage, but this has not been tested. Although this has not been explicitly tested, other physico‐chemical conditions may alter the response to elevated CO_2_. Notably the availability of nutrients can affect pH, thus the availability of CO_2_, and growth rate or peak biomass, thus the drawdown and competition for CO_2_.

In freshwater phytoplankton, it has been demonstrated that differences in the capacity to uptake and utilize CO_2_ between major phytoplankton taxa can lead to predictable changes in their competitive ability in response to rising atmospheric CO_2_ (Low‐Décarie, Fussmann, & Bell, 2011). In contrast to most freshwater systems, marine environments have very little dissolved inorganic carbon (DIC) available as pCO_2_, requiring specific investigation of the differences between major taxonomic groups in their growth response to CO_2_ and predictability of associated shifts in community composition in marine systems.

This study aims to test whether information on differences between major taxonomic groups of marine phytoplankton are sufficient to predict the effect of increasing CO_2_ on their capacity to compete. Taxonomic groups that are more efficient at concentrating and utilizing CO_2_, such as the genus *Synechococcus*, would be expected to have a smaller growth response and a decreased ability to outcompete taxonomic groups that are less efficient at concentrating and utilizing CO_2_, such as chlorophytes, under elevated CO_2_ all else being equal. In contrast, taxonomic groups with specific functional traits, such as calcification in the coccolithophores, maybe deleteriously affected by the increase in CO_2_. We measured how the growth rates, paired competitive abilities, and compositions of a community of seven phytoplankton species belonging to four major taxa (cyanobacteria, chlorophytes, diatoms, and coccolithophores) responded to an increase in CO_2_. We test how robust our findings are to changes in the growth regime and nutrient concentration by replicating this study in both high‐nitrogen batch cultures and lower‐nitrogen semicontinuous cultures.

## 2 MATERIALS AND METHODS

### 2.1 Phytoplankton cultures

We studied seven species from four dominant marine phytoplankton groups: the cyanobacterium *Synechococcus* sp. (CCMP 2370, mean ± range 1.2 ± 0.4 μm diameter, Hughes, Franklin, & Malin, 2011); the chlorophytes *Dunaliella tertiolecta* (CCMP 1320, 11 ± 1 μm diameter) and *Prasinococcus capsulatus* (CCMP 1194, 4.5 ± 1 μm diameter); the diatoms *Phaeodactylum tricornutum* (CCMP 2561, ~21 μm by 3.5 μm) and *Thalassiosira weissflogii* (CCMP 1051, 15 ± 10 μm diameter); and finally, the calcifying coccolithophores *E. huxleyi* (PLY 1516, 4 ± 1 μm diameter) and *Coccolithus pelagicus* (PLY 183, 25 ± 15 μm diameter). Each major taxon, with the exception of the cyanobacteria, was represented by two species, each selected based on being ecologically relevant and being clearly identifiable through morphological features visible under microscopy. There was a large difference in size between the pairs of species from a group (25% difference for diatoms, 240% for chlorophytes, and 625% for coccolithophores) so that there was an overlap in cell size (and associated surface area to volume ratio) between all groups, with the exception of the smaller cyanobacterium.

### 2.2 Growth conditions

All phytoplankton species were grown in Enriched Seawater Artificial Water (ESAW) (Berges, Franklin, & Harrison, 2001) at 15 ± 0.1°C, under a 12:12 hr light:dark cycle at irradiance levels of 250.1 ± 4.5 μmol m^−2^ s^−1^. All cultures were kept in suspension on platform rockers set to 70 rotations per minute (rpm) in two CO_2_‐controlled growth chambers (Adaptis CMP6010, Conviron, Canada). The relative humidity levels across both chambers averaged 82 ± 6.3%. One chamber simulated ambient CO_2_ conditions (506 ± 6 μatm, local conditions lead to higher than the global average of 400 μatm) and the other future CO_2_ conditions (1,000 ± 7 μatm) predicted for the year 2100 (IPCC, 2014). The growth medium was first equilibrated in the chamber conditions for a period of 3 days prior to inoculating the phytoplankton in test tubes. Each of the phytoplankton species was acclimated for a period of 2 weeks (one transfer cycle) before experimentation by maintaining stock cultures in each CO_2_ and nutrient treatment. Stock cultures were maintained in 50 ml of media within 150 ml glass flasks stoppered with air‐permeable foam caps and were maintained in exponential growth with weekly 1:10 dilution.

### 2.3 Growth and competition experiments

All experiments were conducted in 8 ml of medium in 15 ml test tubes fitted with polyurethane foam stoppers and initiated with a starting inoculation of 1 × 10^5^ cells/ml, taken from each of the single species cultures pre‐acclimated under each nutrient and CO_2_ regime. All cultures remained in exponential growth throughout each of the 5‐day experiments. Triplicates of seven pure (individual species), 21 pairwise mixtures and one full community culture were inoculated for each condition (CO_2_ treatment and culture regime). To account for any chamber effects, the experiments were repeated switching the CO_2_ treatment between chambers and repeated twice in each chamber configuration (total replication if pooling across chambers is 12, for a total of 1,392 experimental cultures).

To test the effect of growth regime on the link between growth and community response, all experiments were conducted in two growth regimes that differed in nutrient concentration and maximal cell density. In the high‐nitrogen batch culture regime, the standard ESAW medium (882 μmol/L nitrogen as in F/2 medium; Jutson, Pipe, & Tomas, 2016) was used and the culture was tracked for 5 days without replenishing the medium. Long‐term culturing of species in our culture collection was carried out using ESAW ensuring acclimation to this media. In the lower‐nitrogen semicontinuous culture regime, the nitrogen concentration in the medium was 55 μmol/L N, all other nutrients were at the same levels as in the high‐nitrogen batch culture regime (i.e., F/2 medium) and all cultures were replenished daily over the 5‐day period with 1 ml of fresh lower nitrogen media (1:8 replenishment).

### 2.4 Quantification

To track changes in the cultures throughout the experiment, samples were taken daily from all test tubes. Samples from both the single species cultures and the control tubes containing only the growth media (blanks) were taken on the final day of each run to measure both pH and alkalinity (Snoeyink & Jenkins, 1980) to permit the estimation of the level of dissolved CO_2_ using the CO_2_Calc application (Robbins, Hansen, Kleypas, & Meylan, 2010). Pure culture cell densities were measured daily from the day of inoculation until the final day through either haemocytometry or flow cytometry for the calculation of their respective growth rates. Fresh samples of all other pure cultures were passed directly through a flow cytometer (Accuri C6, BD Bioscience, USA). For flow cytometry, a protocol with a medium flow rate of 0.583 μl/s and a total of 10,000 events recorded was used following the production of a template file using forward and side scatter profiles to standardize the cell counts. Due to flow cytometer use and set up, cyanobacteria could not be counted on the flow cytometer. Cyanobacteria samples were immediately fixed with Lugol's solution (1% final concentration) and injected into a haemocytometer slide and counted using microscopy. Samples from each of the competition mixtures were also immediately fixed with Lugol's solution and stored at 4°C until they were counted via microscopy, whereby the abundance of each species comprising the mixtures were counted using a total minimum count of 400 cells.

### 2.5 Statistical analysis

To measure the response of growth to treatments, exponential growth rates of each culture were calculated as the ratio of the natural‐log of cell densities over the 5‐day experimental period. Predicted competition coefficients were calculated from pure culture growth rate, while realized competition coefficients were calculated from changes in frequency (Low‐Décarie et al., 2011). A change in the sign of the competition coefficient indicates a change in competitive dominance, but a change in the competition coefficient that does not alter the sign indicates a change in the speed of competitive exclusion. Predicted (*p*) competition coefficient of species 1 (*c*_1_) was calculated as the difference of its growth rate with the growth rate of a competing species (*r*_2_) standardized by the growth rate of the entire competing community (*r*_community_):

(1) $$\text{Predicted}\;\text{competition}\;\text{coefficient}\;c_{1p}=\frac{r1-r2}{r_{\mathrm{community}}}$$

Realized (*r*) competition coefficients (Equation 2) were calculated from the change in the frequency (aka. relative frequency, *f*) of each species through time accounting for the growth of the community overall (number of generations across the community *g*_community_):

(2) $$\text{Realized}\;\text{competition}\;\text{coefficient}\;c_{1r}=\frac{1}{g_{\mathrm{community}}}\text{ln}\left(\frac{\frac{f_{1}\text{final}}{f_{2}\text{final}}}{\frac{f_{1}\text{initial}}{f_{2}\text{initial}}}\right)$$

In the full community of seven species, *f*_2_ was the frequency of all other species combined. Phytoplankton responses to CO_2_ were calculated as the difference between growth rates or competition coefficients in ambient and high CO_2_ treatments. The measured competition coefficient, based on change in frequency, integrates any effect of one species on the frequency of another species (whether through limited growth through resource competition or through some other ecological interactions, including facilitation or allelopathy).

The response of dissolved CO_2_ in cultures, growth rates, and competition coefficients were assessed using an analysis of variance (ANOVA), in which the main effects and interactions of CO_2_ treatment, culture regime, and major taxonomic group were tested, the main effect of species was also included. When needed, individual ANOVAs were conducted for the response of each separate taxonomic group, with the main effects and interactions of CO_2_ treatment, culture regime, and species. Full community competitions were assessed using a multivariate analysis of variance (MANOVA) when phytoplankton were grouped by major taxa, and when grouped by species. Predictions of the response of competition to CO_2_ from the response of growth rates was assessed by averaging the competition and growth response by species and fitting a linear regression.

In text, values are expressed as means ±1 standard deviation. Analyses were conducted within the R statistical coding package (R Development Core Team, 2013) and figures were produced in Microsoft Excel.

## 3 RESULTS

### 3.1 Dissolved CO_2_ concentration

CO_2_ concentration was controlled in the atmosphere and some CO_2_ drawdown was expected in growing cultures, so the effect of treatment on DIC concentration needed to be tested. Changes in atmospheric CO_2_ concentrations caused the expected changes in pCO_2_ (Figure S1, 310.7 ± 23.5–711.2 ± 94.4 μatm, *F*_1,336_ = 10,427, *p* < .001) and pH (from 8.15 ± 0.02 to 7.85 ± 0.03). The concentration of nitrogen in each culture regime also had an effect on the pH (8.02 ± 0.16 in high‐N vs. 7.88 ± 0.14 in lower nitrogen) and thus pCO_2_ (19.0 ± 20.0 μatm higher in low‐nitrogen, *F*_1,336_ = 26.7, *p* < .001). All cultures drew down on average 140.4 ± 117.1 μatm of CO_2_ compared to blank media over the 5‐day experiment, but the difference between ambient and high CO_2_ treatments remained throughout the growth of the cultures (average 198.3 ± 76.2 μatm difference, *F*_1,336_ = 2,389 , *p* < .001, Table S1 for drawdown for each species).

### 3.2 Growth response

**Figure 1:**
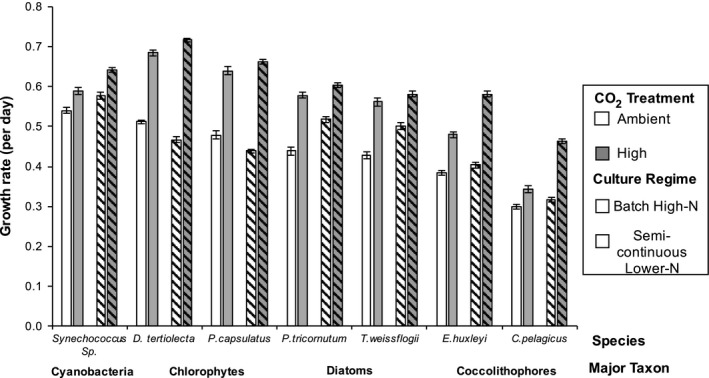
Phytoplankton growth rates across CO_2_ and culture regimes. Plain bars are batch high nitrogen conditions (high and low CO_2_), bars with stripes are semicontinuous lower nitrogen conditions (high and low CO_2_), white bars are ambient CO_2_ (~500 μatm), and shaded bars are high CO_2_ (~1,000 μatm). Each bar shows the mean values with ±1 standard deviation (*N* = 12). All species had a higher growth rate in high CO_2_ compared to low CO_2_ independent of culture regime

The effect of an increase in atmospheric CO_2_ on phytoplankton growth rates was assessed. Growth rates recorded for each phytoplankton culture between chambers did not differ (Table S2), indicating that there was no confounding chamber effect upon their responses, so assays in each chamber were treated as replicates. Growth rates of all species increased with high CO_2_ independent of culture regime, where on average an increase of 0.12 ± 0.07 day^−1^ was observed in phytoplankton exposed to high CO_2_ compared to ambient conditions (Figure 1, *F*_1,336_ = 106, *p* < .001). The scale of this change was taxa‐ and culture‐regime dependent (*F*_3,336_ = 240, *p* < .001). Chlorophytes had the largest average increase in growth rate between CO_2_ treatments of 0.20 ± 0.04 day^−1^, whereas *Synechococcus* had the smallest increase of 0.06 ± 0.01 day^−1^. Species within each major taxon also differed in their response to high CO_2_ (Table S3).

### 3.3 Pairwise competitions

**Figure 2:**
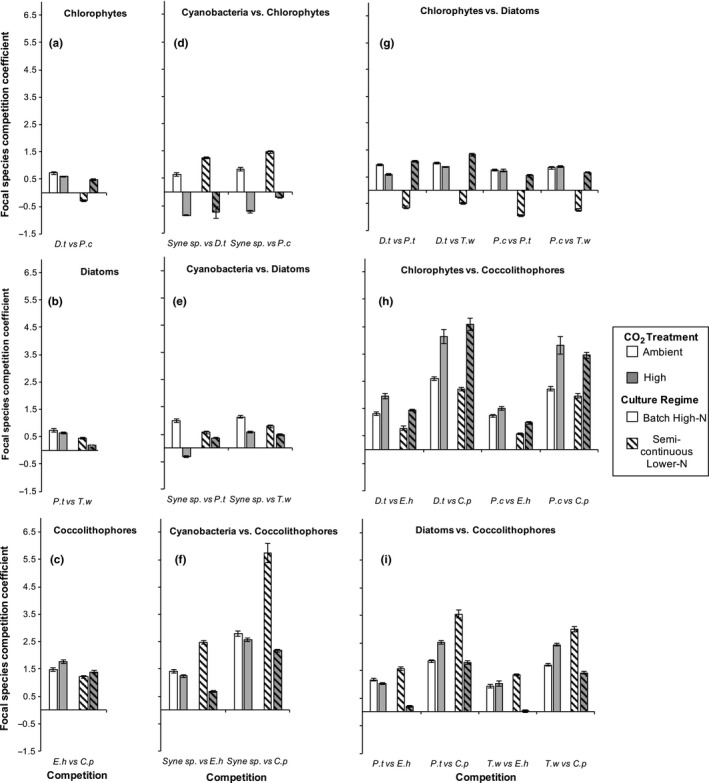
Competition coefficient in all pairwise competitions. (a–c) Competitions between members belonging to the same taxonomic group, (d–f) competitions where the focal competitor was *Synechococcus* sp. (*Syne* sp.; cyanobacterium), (g–h) competitions with chlorophytes (*Dunaliella tertiolecta—D.t‐* and *Prasinococcus capsulatus—P.c‐*) as focal competitors, (f) comparisons with diatoms as focal competitor species (*Phaeodactylum tricornutum—P.t‐, Thalassiosira weissflogii—T.w)*. The coccolithophores are shown in competition but not as focal species (*Emiliania huxleyi—E.h‐* and *Coccolithus pelagicus—C.p‐)*. Statistics and legend match Figure 1: shading (high CO_2_), stripes (semicontinuous lower nitrogen), each bar shows the mean values with ±1 standard deviation (*N* = 12). High CO_2_ decreases the competitive ability of *Synechococcus* while mostly increasing the competitive ability of chlorophytes

The effect of increasing atmospheric CO_2_ upon the competitive ability of each species within each of the pairwise competitions was assessed across both culture regimes. There was a log‐linear change in species frequency in each competition culture (Figures S2 and S3). On average, the response of competitions between species of the same taxonomic group was smaller than the average change in competition coefficient between species of different major groups (same group: average absolute change of 0.28 ± 0.23, Figure 2a–c, different group: average absolute change = 1.02 ± 0.48, *t*_144_ = 11.78, *p* < .001, Figure 2d–i). The competitive ability of *Synechococcus* declined under the high CO_2_ treatment independent of the taxonomic group it was competing with (Figure 2d–f, average decrease of 1.24 ± 0.98 in competition coefficient, *F*_1,288_ = 1,386, *p* < .001). The chlorophytes on average competed better with increased CO_2_ levels (Figure 2d,g–h, average increase of 1.1 ± 0.76 in competition coefficient, *F*_1,480_ = 2,118, *p* < .001), however, a partial reversal of this trend was observed under batch high‐nitrogen conditions when competing against diatoms (*F*_2,480_ = 96, *p* < .001). The competitive response of the diatoms and coccolithophores to elevated CO_2_ conditions was dependent on the competing taxonomic group (Figure 2e–f, diatom: average decrease of 0.32 ± 0.92 in competition coefficient, *F*_3,480_ = 518, *p* < .001, coccolithophore: average decrease of 0.28 ± 0.41, *F*_3,480_ = 183.7, *p* < .001) and was species‐specific when the coccolithophores competed with the diatoms (*F*_2,192_ = 18.2, *p* < .001).

### 3.4 Full community competitions

**Figure 3:**
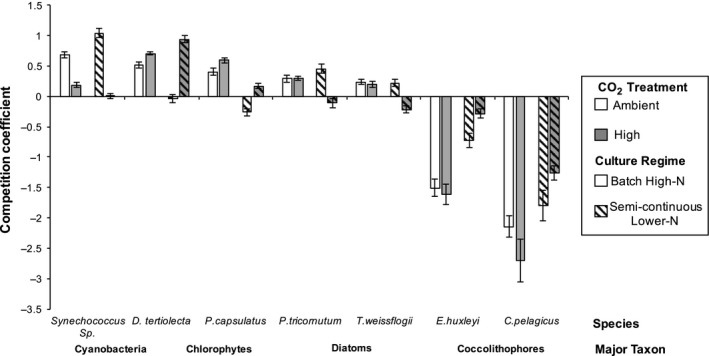
Full community competition coefficients. Elevated CO_2_ decreases the competitive ability of *Synechococcus* and the diatoms, increases the competitive ability of the chlorophytes, but did not exhibit an overall effect on the coccolithophores unless interacting with culture regime, where their competitive ability within semicontinuous‐lower nitrogen cultures increased but decreased in batch high‐nitrogen cultures. Matches previous figures: gray (high CO_2_), stripes (semicontinuous lower nitrogen), bar value (mean), and error bars (one standard deviation *N *= 12)

The assembled community was not stable (extinctions were eventually expected but not observed) and there was a log‐linear change in species frequency in the community comprising all seven species (Figure S4). These competitive dynamics in the full community were also altered by the CO_2_ treatment. The competitive ability of *Synechococcus* decreased the most when CO_2_ levels increased (Figure 3; average decrease of 0.77 ± 0.29 in competition coefficient, *F*_1,48_ = 2,197.6, *p* < .001), although it remained a dominant competitor with a positive competition coefficient, and the diatoms also decreased, although to a lesser extent (average decrease of 0.20 ± 0.21 in competition coefficient, *F*_1,96_ = 194.4, *p* < .001). The chlorophytes, on the other hand, were the only taxon which increased their competitive abilities (average increase of 0.44 ± 0.34 in competition coefficient, *F*_1,96_ = 107.1, *p* < .001) and the response of the coccolithophores was found to be species‐specific (Table S3), where *E. huxleyi's* competition coefficient increased by on average 0.19 ± 0.31 at elevated CO_2_ levels (*p* < .001) but *C. pelagicus* was unaffected (average increase 0.01 ± 0.60 in competition coefficient, *p* > .05).

### 3.5 Predicting phytoplankton community changes

Changes in competition and community dynamics in response to rising CO_2_ were predictable from known difference in the CO_2_‐related taxonomic traits and growth responses to elevated CO_2_. The mean competitive response for each species within pairwise competitions was a good indicator of the competitive ability of each phytoplankton species within the full community (Figure S5), in batch high nitrogen (*R* ^2^ = .75, *p* < .001) and semicontinuous lower nitrogen conditions (*R* ^2^ = .93, *p* < .001).

**Figure 4:**
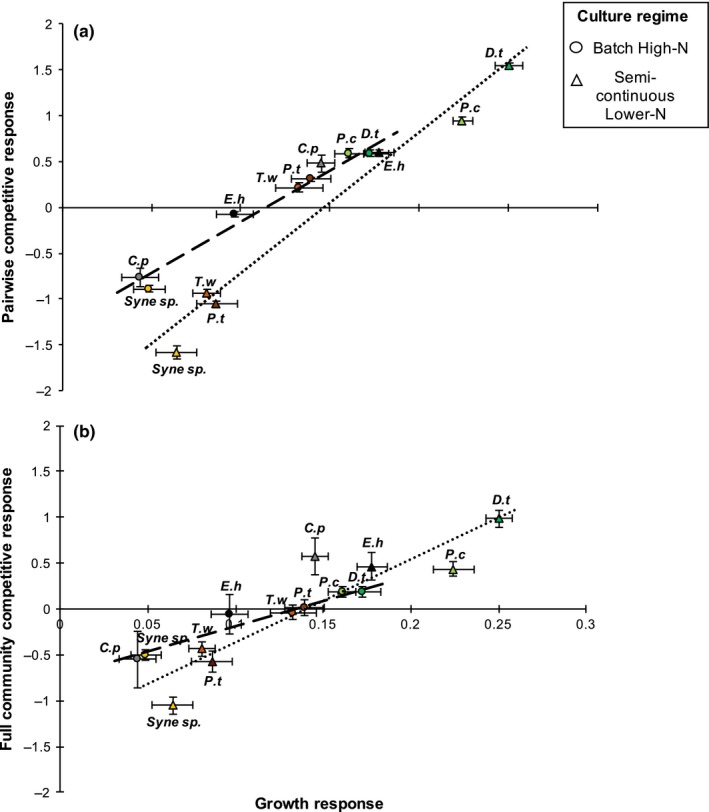
Predicting responses from pure culture growth responses. The growth response of each phytoplankton species was used to predict the average competitive response (a) in pairwise competitions and (b) in the full community comprised of all seven species. Circles are batch high‐nitrogen with dashed line for the regression [(a) *R* ^2^ = .94 and (b) *R* ^2^ = .73] and triangles are semicontinuous lower nitrogen with dotted line for the regression line [(a) *R* ^2^ = .93 and (b) *R* ^2^ = .80]. All values are labeled with the first letter of its genus and species names, and are colored according to both taxonomic group and species. The chlorophytes are shown as dark (*Dunaliella tertiolecta*) and light green (*Prasinococcus capsulatus*), the diatoms as dark (*Phaeodactylum tricornutum*) and light brown (*Thalassiosira weissflogii*), the coccolithophores as black (*Emiliania huxleyi*) and gray (*Coccolithus pelagicus*), and the cyanobacteria are shown as orange (*Synechococcus* sp.). All points displayed are the mean for a species with ±1 standard deviation (*N* = 12). The greatest responses were always exhibited by the chlorophytes, followed by the diatoms (in batch high‐nitrogen conditions) or coccolithophores (in semicontinuous lower nitrogen conditions) and the lowest responses were generally exhibited by *Synechococcus*

Pure culture growth responses were also a good predictor of the overall competitive response of each species within pairwise competitions (Figure 4a) in both batch high nitrogen (*R* ^2^ = .94, *p* < .001) and semicontinuous lower nitrogen conditions (*R* ^2^ = .93, *p* < .001) and of the competitive response in the full community of seven species (Figure 4b), in batch high nitrogen (*R* ^2^ = .73, *p* < .001) and semicontinuous lower nitrogen conditions (*R* ^2^ = .80, *p* < .001).

## 4 DISCUSSION

### 4.1 CO_2_ as a limiting resource

Marine environments are highly dynamic systems in which growth rate is an important parameter for phytoplankton population dynamics. In situ growth rates of communities of phytoplankton range from 0.1 to 3.6 doubling per day (Furnas, 1990). Even in conditions were other factors, such as grazing, pathogens, or maximum total biomass achievable, growth rates will influence dynamics and composition of communities. All phytoplankton species examined in this study had an increased growth rate when exposed to future atmospheric CO_2_ levels across both culture regimes. This contrasts with expectations based on nutrient limitation in natural marine phytoplankton communities, where the main limiting resources are usually nitrogen and iron (Downing, Osenberg, & Sarnelle, 1999). However, they match extensive laboratory experiments looking at growth response to elevated CO_2_ in nutrient‐replete and nutrient‐limited conditions (e.g., meta‐analysis Dutkiewicz et al., 2015). The nitrogen levels on our experiment did not limit growth in either treatment and were high compared to oceanic total nitrogen ranges between 21.9 and 41.0 μmol/L (Guildford & Hecky, 2000), but the lower nitrogen treatment (55 μmol/L N) was within natural range for estuarine and coastal marine ecosystems in which total nitrogen can exceed 150 μmol/L N (Smith, 2006). Biomass of primary producers in marine environments is generally expected to be limited by nitrogen or iron, although there is growing understanding that multiple nutrients, potentially nitrogen and CO_2_, can limit primary production simultaneously (Harpole et al., 2011; Moore et al., 2013). CO_2_ could play a role as a rate‐limiting nutrient, limiting growth rate but not maximal biomass (Low‐Décarie, Fussmann, & Bell, 2014), and thus, may exhibit a stronger limitation role in dynamic marine environments where maximal biomass is rarely reached and dynamics are in part controlled by growth rates. Increased CO_2_ increases primary producer biomass in natural marine phytoplankton communities when other nutrients are added simultaneously (Riebesell et al., 2007) and even in low nutrient concentration and the absence of nutrient addition (Eberlein et al., 2017). However, understanding the role of CO_2_ among other limiting resources requires further experimentation.

### 4.2 Predictability of changes in the composition of communities to a changing environment

Our results on the ecological response to increased CO_2_ in marine phytoplankton align with previous studies of change in competition in freshwater phytoplankton (Low‐Décarie et al., 2011) and with expectation based on the CO_2_‐related physiology of the major taxonomic groups (e.g., Reinfelder, 2011). *Synechococcus,* which has the most efficient uptake and utilization of CO_2_, lose out mostly at the benefit of chlorophytes under high CO_2_ as chlorophytes have likely invested in functional traits not related to carbon utilization and acquisition such as nitrogen scavenging or light harvesting. However, this change in competitive ability did not prevent *Synechococcus* from increasing in frequency in the full community under elevated CO_2_. The change in competition between groups that have more similar capacities for carbon utilization and acquisition (chlorophytes vs. diatoms or diatoms vs. coccolithophores) is less predictable (more species‐specific or depends more on culture regime). Other traits that are not specific to any major groups, such as size and associated surface area to volume ratio, could also influence the expected response to increasing CO_2_, with larger taxa benefiting most from the increase in CO_2_. However, changes in competition under elevated CO_2_ between species of the same group was not consistent, this difference was small when present and it did not align with predictions made by size (smaller species tended to benefit from the increase in CO_2_). Our findings contrast with findings from some studies of in situ natural marine phytoplankton assemblages, including a study showing an increase in the cyanobacterium *Synechococcus* (Paulino et al., 2007) and a decrease in auroxanthin‐containing phytoplankton (diatoms, Yoshimura et al., 2009). In these experiments on natural communities, as in our experiments, growth response is expected to dominate the community dynamics and as these experiments track the response in a bloom elicited through the addition of nutrients. A mesocosm experiment without the addition of nutrients did find a decrease in *Synechococcus* and an increase in major groups of chlorophytes (Crawfurd, Alvarez‐Fernandez, Mojica, Riebesell, & Brussaard, 2017). Difference in the response of natural assemblages and those of simplified laboratory communities are not surprising, and can be explained by factors including interactions with other treatments (including nutrient addition), changes in communities selective grazer or pathogens in response to the CO_2_ treatment or greater importance of heterotrophic and mixotrophic processes utilizing existing organic carbon stocks. In natural marine communities, competitive dynamics and the response to CO_2_ may also be controlled by the capacity to reach maximal biomass (carrying capacity) or other types of interactions between competitors (e.g., through allelopathy or facilitation). Results of both controlled laboratory studies and natural phytoplankton assemblages are likely to be dependent on the experimental duration due to the interplay of plastic and evolutionary responses of individual species.

### 4.3 Plasticity and evolution

Phytoplankton are able to regulate their CCMs, such that in high CO_2_ conditions they are able to reduce their activity and therefore energy consumption (Giordano, Beardall, & Raven, 2005; Reinfelder, 2011). High CO_2_ exposure is suggested to be accompanied by a down‐regulation of the genes involved with these cellular CCMs (Crawfurd, Raven, Wheeler, Baxter, & Joint, 2011; Van de Waal et al., 2013). Different life stages do not have the same responses to rising CO_2_, for example, haploid and diploid stages in coccolithophores do not have the same response to acidification (Rokitta, John, & Rost, 2012), therefore, a plastic response could arise from a change in life‐history strategy. If these plastic responses in gene regulation are not captured by the time scale of our experiments (5 days) and differ markedly between taxa, they could affect the predictability of the changes in competitions under elevated CO_2_.

Marine phytoplankton may eventually adapt to higher CO_2_ concentrations. As for plasticity, potential differences in the rate of adaptation or scale of adaptive gains between major taxonomic groups or species may alter the expected changes in competition. However, freshwater phytoplankton were not found to specifically adapt to elevated CO_2_ (Collins & Bell, 2004, 2006; Collins, Sultemeyer, & Bell, 2006; Low‐Decarie, Jewell, Fussmann, & Bell, 2013), although prolonged exposure to elevated CO_2_ can lead to a decreased ability to grow under lower CO_2_ (Collins & Bell, 2004). These findings in freshwater phytoplankton may not be transferable to marine algae. In calcifying phytoplankton, the change in pH associated with higher CO_2_ concentrations could be expected to act as a strong selective pressure leading to faster evolution in this group (Collins, Rost, & Rynearson, 2014). The coccolithophore *E. huxleyi*, a calcifying phytoplankton, has been shown to adapt to high CO_2_ conditions in marine systems within 500 generations (Lohbeck, Riebesell, Collins, & Reusch, 2013; Lohbeck, Riebesell, & Reusch, 2012). Another coccolithophore species, *Gephyrocapsa oceanica*, did evolve under high CO_2_, although it is not clear that observed changes were an adaptive response to CO_2_ (Tong, Gao, & Hutchins, 2018). Beyond calcifying phytoplankton, the evolutionary implications of elevated CO_2_ for marine phytoplankton, and thus its potential effect on the predictability of changes in competition and community composition, is not well resolved. An experiment with the cyanobacterium *Trichodesmium*, a globally important diazotroph, showed adaptation to elevated CO_2_ conditions when maintained at high CO_2_, but it was not CO_2_ specific, with lines evolved at elevated‐CO_2_ growing better than the ambient selected lines independent of CO_2_ concentrations (Walworth, Lee, Fu, Hutchins, & Webb, 2016). To test for the impact of adaptation on the predicted changes in competitive dynamics under elevated CO_2_, the experiment presented in this study could be repeated with high CO_2_‐adapted lines of each major taxonomic group if the required long‐term selection experiments are conducted.

### 4.4 Implications of changes in community composition

In addition to differing in the carbon acquisition and use, the major taxonomic groups of phytoplankton have different ecological roles. On average, diatoms have some of the fastest sinking rates (Fahnenstiel et al., 1995) and play a major role in exporting primary productivity from the euphotic zone. Coccolithophores release CO_2_ through calcification and decrease the DIC pool so that the increase in coccolithophores with higher CO_2_, seen for at least the species from this study (*E. huxleyi* which is most abundant and widespread coccolithophores in the ocean), could lead to a feedback and a further increase in dissolved CO_2_ concentration. The association of CO_2_ response and ecological role of marine phytoplankton taxonomic groups lead models to suggest that the repercussions of change in the community composition for ecological function will exceed the effects of warming and reduced nutrient supply arising from global change (Dutkiewicz et al., 2015).

Our laboratory experiments and the resulting predictions of major ecosystem level repercussions from the change in phytoplankton communities with rising CO_2_ ignore numerous ecological complexities. In addition to the limitation already raised about high‐nutrient concentrations and a small set of laboratory strains, further caveats include that the natural phytoplankton communities are embedded in complex food webs, in which each trophic level, or even each species, may respond to ocean acidification, and thus modulate the response of phytoplankton to rising CO_2_. Rising CO_2_ could thus still affect phytoplankton in ways that do not depend on the capacity of major taxonomic groups of phytoplankton to uptake and utilize CO_2_. In addition, ocean acidification is only one of many current anthropogenic changes affecting our world's oceans. Nonetheless, that the change in community composition with rising CO_2_ of a functionally diverse community of phytoplankton can be predicted from growth response of individual species suggests that some useful inferences can be made from the study of individual taxa for the prediction of how marine communities will respond to global changes.

## CONFLICT OF INTEREST

None declared.

## AUTHORS’ CONTRIBUTIONS

Jacob Pardew designed and conducted the experiment, analyzed the results, and drafted portions of the manuscript. Macarena Blanco Pimentel conducted a pilot experiment and contributed to the experimental design. Etienne Low‐Décarie advised on the design of the experiment, its analysis, and drafted portions of the manuscript. All authors contributed to the review and editing of the complete manuscript.

## DATA ACCESSIBILITY

Data and analysis script are available on Zenodo (https://doi.org/10.5281/zenodo.1172665).

## References

[ece33971-bib-0001] Almén, A. K. , Vehmaa, A. , Brutemark, A. , Bach, L. , Lischka, S. , Stuhr, A. , … Engström‐Öst, J. (2016). Negligible effects of ocean acidification on *Eurytemora affinis* (Copepoda) offspring production. Biogeosciences, 13, 1037–1048. https://doi.org/10.5194/bg-13-1037-2016

[ece33971-bib-0002] Badger, M. R. , & Price, G. D. (2003). CO_2_ concentrating mechanisms in cyanobacteria: Molecular components, their diversity and evolution. Journal of Experimental Botany, 54, 609–622. https://doi.org/10.1093/jxb/erg076 12554704 10.1093/jxb/erg076

[ece33971-bib-0003] Berges, J. A. , Franklin, D. J. , & Harrison, P. J. (2001). Evolution of an artificial seawater medium: Improvements in enriched seawater, artificial water over the last two decades. Journal of Phycology, 37, 1138–1145. https://doi.org/10.1046/j.1529-8817.2001.01052.x

[ece33971-bib-0004] Bermúdez, R. , Winder, M. , Stuhr, A. , Almén, A.‐K. , Engström‐Öst, J. , & Riebesell, U. (2016). Effect of ocean acidification on the structure and fatty acid composition of a natural plankton community in the Baltic Sea. Biogeosciences, 13, 6625–6635. https://doi.org/10.5194/bg-13-6625-2016

[ece33971-bib-0005] Brewer, P. , Fabry, Vi. , Hilmi, K. , Jung, S. , Poloczanska, E. , & Sundby, S. (2014). The Ocean. *Fifth Assessment Report—Impacts, Adaptation and Vulnerability—IPCC* (pp. 1–51).

[ece33971-bib-0006] Collins, S. S. , & Bell, G. (2004). Phenotypic consequences of 1,000 generations of selection at elevated CO_2_ in a green alga. Nature, 431, 566–569. https://doi.org/10.1038/nature02945 15457260 10.1038/nature02945

[ece33971-bib-0007] Collins, S. , & Bell, G. (2006). Evolution of natural algal populations at elevated CO_2_ . Ecology Letters, 9, 129–135. https://doi.org/10.1111/j.1461-0248.2005.00854.x 16958877 10.1111/j.1461-0248.2005.00854.x

[ece33971-bib-0008] Collins, S. , Rost, B. , & Rynearson, T. A. (2014). Evolutionary potential of marine phytoplankton under ocean acidification. Evolutionary Applications, 7, 140–155. https://doi.org/10.1111/eva.12120 24454553 10.1111/eva.12120 PMC3894903

[ece33971-bib-0009] Collins, S. , Sultemeyer, D. , & Bell, G. (2006). Changes in C uptake in populations of *Chlamydomonas reinhardtii* selected at high CO_2_ . Plant, Cell and Environment, 29, 1812–1819. https://doi.org/10.1111/j.1365-3040.2006.01559.x 10.1111/j.1365-3040.2006.01559.x 16913870

[ece33971-bib-0010] Crawfurd, K. J. , Alvarez‐Fernandez, S. , Mojica, K. D. A. , Riebesell, U. , & Brussaard, C. P. D. (2017). Alterations in microbial community composition with increasing fCO_2_: A mesocosm study in the eastern Baltic Sea. Biogeosciences, 14, 3831–3849. https://doi.org/10.5194/bg-14-3831-2017

[ece33971-bib-0011] Crawfurd, K. J. , Raven, J. A. , Wheeler, G. L. , Baxter, E. J. , & Joint, I. (2011). The response of *Thalassiosira pseudonana* to long‐term exposure to increased CO_2_ and decreased pH. PLoS ONE, 6, 1–9. 10.1371/journal.pone.0026695 PMC3203894 22053201

[ece33971-bib-0012] Downing, J. A. , Osenberg, C. W. , & Sarnelle, O. (1999). Meta‐analysis of marine nutrient‐enrichment experiments: Variation in the magnitude of nutrient limitation. Ecology, 80, 1157–1167. https://doi.org/10.1890/0012-9658(1999)080[1157:MAOMNE]2.0.CO;2

[ece33971-bib-0013] Dutkiewicz, S. , Morris, J. J. , Follows, M. J. , Scott, J. , Levitan, O. , Dyhrman, S. T. , & Berman‐Frank, I. (2015). Impact of ocean acidification on the structure of future phytoplankton communities. Nature Climate Change, 5, 1002–1006. https://doi.org/10.1038/nclimate2722

[ece33971-bib-0014] Eberlein, T. , Wohlrab, S. , Rost, B. , John, U. , Bach, L. T. , Riebesell, U. , & Van De Waal, D. B. (2017). Effects of ocean acidification on primary production in a coastal North Sea phytoplankton community. PLoS ONE, 12, 1–15. 10.1371/journal.pone.0172594 PMC5342202 28273107

[ece33971-bib-0015] Fahnenstiel, G. L. , McCormick, M. J. , Lang, G. A. , Redalje, D. G. , Lohrenz, S. E. , Markowitz, M. , … Carrick, H. J. (1995). Taxon‐specific growth and loss rates for dominant phytoplankton populations from the northern Gulf of Mexico. Marine Ecology Progress Series, 117, 229–239. https://doi.org/10.3354/meps117229

[ece33971-bib-0016] Fu, F. X. , Warner, M. E. , Zhang, Y. , Feng, Y. , & Hutchins, D. A. (2007). Effects of increased temperature and CO_2_ on photosynthesis, growth, and elemental ratios in marine *Synechococcus* and *Prochlorococcus* (cyanobacteria). Journal of Phycology, 43, 485–496. https://doi.org/10.1111/j.1529-8817.2007.00355.x

[ece33971-bib-0017] Furnas, M. J. (1990). In situ growth‐rates of marine‐phytoplankton: Approaches to measurement, community and species growth rates. Journal of Plankton Research, 12, 1117–1151. https://doi.org/10.1093/plankt/12.6.1117

[ece33971-bib-0018] Giordano, M. , Beardall, J. , & Raven, J. A. (2005). CO_2_ concentrating mechanisms in algae: Mechanisms, environmental modulation, and evolution. Annual Review of Plant Biology, 56, 99–131. https://doi.org/10.1146/annurev.arplant.56.032604.144052 10.1146/annurev.arplant.56.032604.144052 15862091

[ece33971-bib-0019] Griggs, D. J. , & Noguer, M. (2002). Climate change 2001: The scientific basis. Contribution of Working Group I to the Third Assessment Report of the Intergovernmental Panel on Climate Change. Weather, 57, 267–269. https://doi.org/10.1256/004316502320517344

[ece33971-bib-0020] Guildford, S. J. , & Hecky, R. E. (2000). Total nitrogen, total phosphorus, and nutrient limitation in lakes and oceans: Is there a common relationship? Limnology and Oceanography, 45, 1213–1223. https://doi.org/10.4319/lo.2000.45.6.1213

[ece33971-bib-0021] Harpole, W. S. , Ngai, J. T. , Cleland, E. E. , Seabloom, E. W. , Borer, E. T. , Bracken, M. E. S. , … Smith, J. E. (2011). Nutrient co‐limitation of primary producer communities. Ecology Letters, 14, 852–862. https://doi.org/10.1111/j.1461-0248.2011.01651.x 21749598 10.1111/j.1461-0248.2011.01651.x

[ece33971-bib-0022] Hopkinson, B. M. , Dupont, C. L. , Allen, A. E. , & Morel, F. M. M. (2011). Efficiency of the CO_2_‐concentrating mechanism of diatoms. Proceedings of the National Academy of Sciences of the United States of America, 108, 3830–3837. https://doi.org/10.1073/pnas.1018062108 21321195 10.1073/pnas.1018062108 PMC3054024

[ece33971-bib-0023] Hughes, C. , Franklin, D. J. , & Malin, G. (2011). Iodomethane production by two important marine cyanobacteria: *Prochlorococcus marinus* (CCMP 2389) and *Synechococcus* sp. (CCMP 2370). Marine Chemistry, 125, 19–25. https://doi.org/10.1016/j.marchem.2011.01.007

[ece33971-bib-0024] Hutchins, D. A. , Fu, F.‐X. , Webb, E. A. , Walworth, N. , & Tagliabue, A. (2013). Taxon‐specific response of marine nitrogen fixers to elevated carbon dioxide concentrations. Nature Geoscience, 6, 790–795. https://doi.org/10.1038/ngeo1858

[ece33971-bib-0025] IPCC (2014). Observations: Ocean In Intergovernmental Panel on Climate Change (Ed.), Climate change 2013—The physical science basis (pp. 255–316). Cambridge, UK: Cambridge University Press.

[ece33971-bib-0026] Jutson, M. G. S. , Pipe, R. K. , & Tomas, C. R. (2016). The cultivation of marine phytoplankton In Tsaloglou M.‐N. (Ed.), Microalgae: Current research and applications (pp. 11–26). Poole, UK: Caister Academic Press https://doi.org/10.21775/9781910190272

[ece33971-bib-0027] Kroeker, K. J. , Kordas, R. L. , Crim, R. N. , & Singh, G. G. (2010). Meta‐analysis reveals negative yet variable effects of ocean acidification on marine organisms. Ecology Letters, 13, 1419–1434. https://doi.org/10.1111/j.1461-0248.2010.01518.x 20958904 10.1111/j.1461-0248.2010.01518.x

[ece33971-bib-0028] Lohbeck, K. T. , Riebesell, U. , Collins, S. , & Reusch, T. B. H. (2013). Functional genetic divergence in high CO_2_ adapted *Emiliania huxleyi* populations. Evolution, 67, 1892–1900. https://doi.org/10.1111/j.1558-5646.2012.01812.x 23815647 10.1111/j.1558-5646.2012.01812.x

[ece33971-bib-0029] Lohbeck, K. T. , Riebesell, U. , & Reusch, T. B. H. (2012). Adaptive evolution of a key phytoplankton species to ocean acidification. Nature Geoscience, 5, 1–6.

[ece33971-bib-0030] Low‐Décarie, E. , Fussmann, G. F. , & Bell, G. (2011). The effect of elevated CO_2_ on growth and competition in experimental phytoplankton communities. Global Change Biology, 17, 2525–2535. https://doi.org/10.1111/j.1365-2486.2011.02402.x

[ece33971-bib-0031] Low‐Décarie, E. , Fussmann, G. F. , & Bell, G. (2014). Aquatic primary production in a high‐CO_2_ world. Trends in Ecology & Evolution, 29, 1–10. 24631287 10.1016/j.tree.2014.02.006

[ece33971-bib-0032] Low‐Decarie, E. , Jewell, M. D. , Fussmann, G. F. , & Bell, G. (2013). Long‐term culture at elevated atmospheric CO_2_ fails to evoke specific adaptation in seven freshwater phytoplankton species. Proceedings of the Royal Society B: Biological Sciences, 280, 20122598–20122598. https://doi.org/10.1098/rspb.2012.2598 23303540 10.1098/rspb.2012.2598 PMC3574323

[ece33971-bib-0033] Meyer, J. , & Riebesell, U. (2015). Reviews and syntheses: Responses of coccolithophores to ocean acidification: A meta‐analysis. Biogeosciences, 12, 1671–1682. https://doi.org/10.5194/bg-12-1671-2015

[ece33971-bib-0034] Moore, C. M. , Mills, M. M. , Arrigo, K. R. , Berman‐Frank, I. , Bopp, L. , Boyd, P. W. , … Ulloa, O. (2013). Processes and patterns of oceanic nutrient limitation. Nature Geoscience, 6, 701–710. https://doi.org/10.1038/ngeo1765

[ece33971-bib-0035] Paulino, A. I. , Egge, J. K. , & Larsen, A. (2007). Effects of increased atmospheric CO_2_ on small and intermediate sized osmotrophs during a nutrient induced phytoplankton bloom. Biogeosciences Discussions, 4, 4173–4195. https://doi.org/10.5194/bgd-4-4173-2007

[ece33971-bib-0036] Price, G. D. , Badger, M. R. , Woodger, F. J. , & Long, B. M. (2008). Advances in understanding the cyanobacterial CO_2_‐concentrating‐ mechanism (CCM): Functional components, Ci transporters, diversity, genetic regulation and prospects for engineering into plants. Journal of Experimental Botany, 59, 1441–1461. https://doi.org/10.1093/jxb/erm112 10.1093/jxb/erm112 17578868

[ece33971-bib-0037] R Development Core Team (2013). R: A language and environment for statistical computing. Vienna, Austria: R Foundation for Statistical Computing Retrieved from http://www.R-project.org/

[ece33971-bib-0038] Reinfelder, J. R. (2011). Carbon concentrating mechanisms in eukaryotic marine phytoplankton. Annual Review of Marine Science, 3, 291–315. https://doi.org/10.1146/annurev-marine-120709-142720 10.1146/annurev-marine-120709-142720 21329207

[ece33971-bib-0039] Riebesell, U. , Schulz, K. G. , Bellerby, R. G. J. , Botros, M. , Fritsche, P. , Meyerhöfer, M. , … Zöllner, E. (2007). Enhanced biological carbon consumption in a high CO_2_ ocean. Nature, 450, 545–548. https://doi.org/10.1038/nature06267 17994008 10.1038/nature06267

[ece33971-bib-0040] Robbins, L. L. , Hansen, M. E. , Kleypas, J. A. , & Meylan, S. C. (2010). CO_2_calc: A user‐friendly seawater carbon calculator for Windows, Mac OS X, and iOS (iPhone). Reston, VA: U.S. Geological Survey.

[ece33971-bib-0041] Rokitta, S. D. , John, U. , & Rost, B. (2012). Ocean acidification affects redox‐balance and ion‐homeostasis in the life‐cycle stages of *Emiliania huxleyi* . PLoS ONE, 7, e52212 https://doi.org/10.1371/journal.pone.0052212 23300616 10.1371/journal.pone.0052212 PMC3530605

[ece33971-bib-0042] Smith, V. H. (2006). Responses of estuarine and coastal marine phytoplankton to nitrogen and phosphorus enrichment. Limnology and Oceanography, 51, 377–384. https://doi.org/10.4319/lo.2006.51.1_part_2.0377

[ece33971-bib-0043] Snoeyink, V. , & Jenkins, D. (1980). Water chemistry, 1st ed New York, NY: John Wiley & Sons.

[ece33971-bib-0044] Tong, S. , Gao, K. , & Hutchins, D. A. (2018). Adaptive evolution in the coccolithophore *Gephyrocapsa oceanica* following 1000 generations of selection under elevated CO_2_ . Global Change Biology, 38, 42–49. 10.1111/gcb.14065 29356310

[ece33971-bib-0045] Tortell, P. D. (2000). Evolutionary and ecological perspectives on carbon acquisition in phytoplankton. Limnology and Oceanography, 45, 744–750. https://doi.org/10.4319/lo.2000.45.3.0744

[ece33971-bib-0046] Tortell, P. D. , Payne, C. D. , Li, Y. , Trimborn, S. , Rost, B. , Smith, W. O. , … DiTullio, G. R. (2008). CO_2_ sensitivity of Southern Ocean phytoplankton. Geophysical Research Letters, 35, L04605.

[ece33971-bib-0047] Van de Waal, D. B. , John, U. , Ziveri, P. , Reichart, G. J. , Hoins, M. , Sluijs, A. , & Rost, B. (2013). Ocean acidification reduces growth and calcification in a marine dinoflagellate. PLoS ONE, 8, e65987 https://doi.org/10.1371/journal.pone.0065987 23776586 10.1371/journal.pone.0065987 PMC3679017

[ece33971-bib-0048] Walworth, N. G. , Lee, M. D. , Fu, F.‐X. , Hutchins, D. A. , & Webb, E. A. (2016). Molecular and physiological evidence of genetic assimilation to high CO_2_ in the marine nitrogen fixer *Trichodesmium* . Proceedings of the National Academy of Sciences of the United States of America, 113, E7367–E7374. https://doi.org/10.1073/pnas.1605202113 27830646 10.1073/pnas.1605202113 PMC5127367

[ece33971-bib-0400] Wu, Y. , Gao, K. , Riebesell, U. (2010). CO_2_‐induced seawater acidification affects physiological performance of the marine diatom *Phaeodactylum tricornutum* . Biogeosciences, 7, 2915–2923.

[ece33971-bib-0049] Yoshimura, T. , Nishioka, J. , Suzuki, K. , Hattori, H. , Kiyosawa, H. , & Watanabe, Y. W. (2009). Impacts of elevated CO_2_ on phytoplankton community composition and organic carbon dynamics in nutrient‐depleted Okhotsk Sea surface waters. Biogeosciences Discussions, 6, 4143–4163. https://doi.org/10.5194/bgd-6-4143-2009

