# Supplementary material for: Predictable ecological response to rising CO 2 of a community of marine phytoplankton
Source: Ecol Evol. 2018 Apr 2;8(8):4292–302. doi: 10.1002/ece3.3971 (PMC5916311; doi:10.1002/ece3.3971)
Supplement: Supplementary file 1 [file ECE3-8-4292-s001.docx]

**Supplemental information**

Table S1: Analysis of Pure Culture CO2 Drawdown 2

Table S2: Analysis of Pure-Culture Growth Rates 2

Table S3: Analysis of Growth Rates 3

Figure S2: Cyanobacterium Pairwise-Competition Population Dynamics 5

Figure S3: Change in frequency through time in each pairwise competition 7

Figure S4: Population Dynamics in Full Community through time 9

Table S4: Analysis of Full Community Competition 10

Figure 5: Predicting Full Community Competitive Responses from Pairwise Competitive Responses 11

**Figure S1: Dissolved CO_2_ Concentration Across CO_2_ and Culture Regimes.** pCO_2_ for each of the phytoplankton pure cultures and blank test tubes (growth media only). All cultures had higher dissolved CO_2_ levels in high CO_2_ conditions compared to ambient conditions, independent of culture regime. Matches main text figures: plain bars illustrate batch high-nitrogen conditions, bars with stripes indicate semi-continuous lower-nitrogen conditions, white bars show ambient CO_2_ conditions (~500.µatm) and shaded bars are high CO_2_ conditions (~1000.µatm), each bar shows the mean values recorded with ± 1 standard deviation (N=12).

Table S1: Analysis of Pure Culture CO2 Drawdown**.** Analysis of variance (ANOVA) of each pure culture sample’s drawdown of dissolved CO_2_ between ambient and high CO_2_ treatments, culture regime (batch high-N vs. semi-continuous lower-N) and the interaction of both.

****** indicates p<0.01, ******* indicates p<0.001

Table S2: Analysis of Pure-Culture Growth Rates. Analysis of variance (ANOVA) for each of the pure-culture’s growth rates five days.

***** Indicates significance of p<0.05, ****** indicates p<0.01, ******* indicates p<0.001, absense of ***** indicates p>0.05.

Table S3: Analysis of Growth Rates. Analysis of variance (ANOVA) for each of the major taxa’s growth rate over a week.

***** Indicates significance of p<0.05, ****** indicates p<0.01, ******* indicates p<0.001, absense of ***** indicates p>0.05.

Figure S2: Cyanobacterium Pairwise-Competition Population Dynamics**.** Frequencies of each species in all pairwise mixtures with the cyanobacterium. Circles and triangles connected by dotted lines illustrate batch high-nitrogen cultures, and semi-continuous lower-nitrogen cultures are illustrated by plain squares and squares containing crosses. The circles and squares containing crosses illustrate the ambient CO_2_ treatment (~500.µatm), whereas the triangles and plain squares illustrate the high CO_2_ treatments (~1000.µatm). The chlorophytes are shown as dark green (*D. tertiolecta*) and light green (*P. capsulatus*), the diatoms as dark brown (*P. tricornutum*) and light brown (*T. weissflogii*), the coccolithophores as black (*E. huxleyi*) and grey (*C. pelagicus*), and the cyanobacteria as orange (*Synechococcus* sp.). The panels show all competitions between the cyanobacteria and the (a-c) chlorophytes, (c-d) diatoms, and (e-f) coccolithophores. Time in days is shown along the *x*-axis, and the frequency of each species shown along the *y*-axis. Data show mean values with ± 1 standard deviation (N=12).

Figure S3: Change in frequency through time in each pairwise competition**.** The chlorophytes are shown as dark green (*D.tertiolecta*) and light green (*P.capsulatus*), the diatoms as dark brown (*P.tricornutum*) and light brown (*T.weissflogii*), the coccolithophores as black (*E.huxleyi*) and grey (*C.pelagicus*), and the cyanobacteria as orange (*Synechococcus sp).* Panels a-h show all competitions where the chlorophytes were the focal competitors, i-l where the diatoms were the focal competitors against the coccolithophores, and m-o the competitions between species belonging to the same taxonomic group. Batch high-nitrogen cultures are represented by circles and triangles connected by dashed lines, squares and crossed-squares connected with full lines are semi-continuous lower-nitrogen. The circles and crossed-squares are the ambient CO_2_ treatment (~500.µatm), whereas the triangles and squares illustrate the high CO_2_ treatments (~1000.µatm). All points displayed are mean values with ± 1 standard deviation (N=12).

Figure S4: Population Dynamics in Full Community through time**.** Samples recorded in the batch high-nitrogen treatment are shown in panel a, and samples recorded in the semi-continuous lower-nitrogen system are shown in panel b). Circles connected by dotted lines illustrate all samples recorded at ambient CO_2_ levels (~500.µatm) and triangles connected by dashed lines illustrate all samples recorded at high CO_2_ levels (~1000.µatm). Each phytoplankton species within the figure are expressed as the first letter of its genus and species names, and are coloured according to both taxonomic group and species. The chlorophytes are shown as dark green (*D.tertiolecta*) and light green (*P.capsulatus*), the diatoms as dark brown (*P.tricornutum*) and light brown (*T.weissflogii*), the coccolithophores as black (*E.huxleyi*) and grey (*C.pelagicus*), and the cyanobacteria as orange (*Synechococcus sp.*)*.* The experimental days are shown along the x axis, and the frequency of each species shown along the y axis. All points displayed are mean values with ± 1 standard deviation (N=12).

Table S4: Analysis of Full Community Competition**.** Multivariate analysis of variance (MANOVA) of competition coefficients involved in the full community competition.

***** Indicates significance of p<0.05, ****** indicates p<0.01, ******* indicates p<0.001, absense of ***** indicates p>0.05.


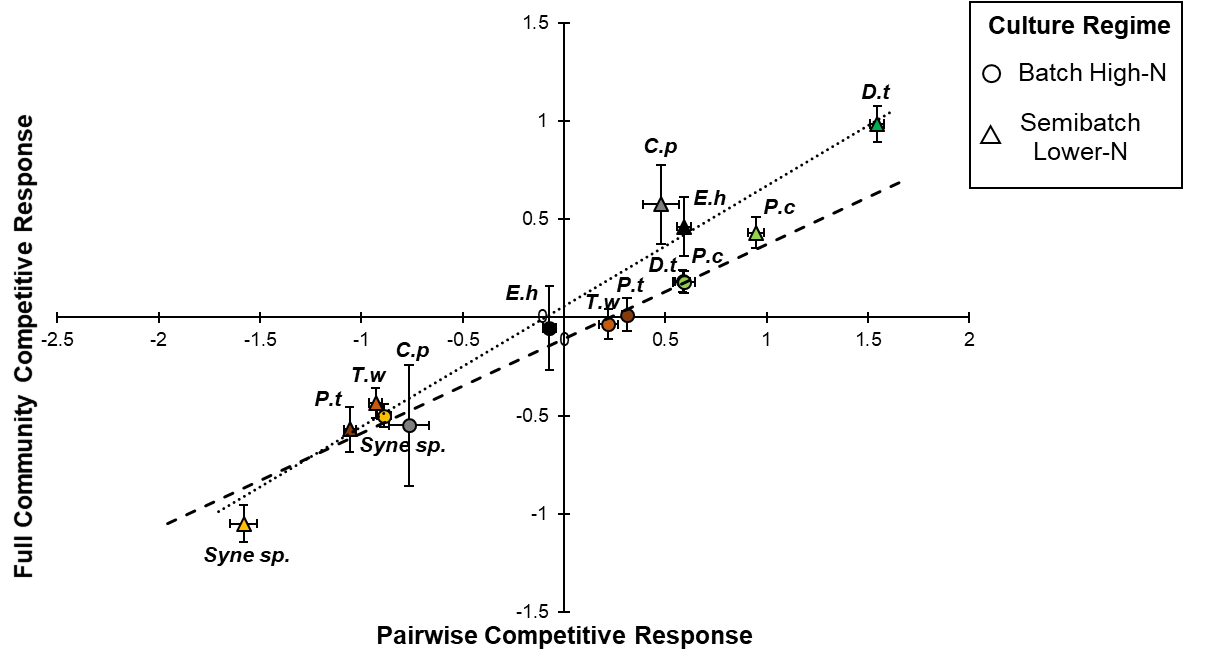


Figure 5: Predicting Full Community Competitive Responses from Pairwise Competitive Responses**.** The average competitive response in pairwise competitions was compared to the competitive response in a full community comprised of 7 species. Circles are batch high-nitrogen conditions with a dashed line for the regression line (R^2^=0.75), whereas triangles are semi-continuous lower-nitrogen conditions with a dotted line for the repression line (R^2^=0.93). All values are labelled with the first letter of its genus and species names, and are coloured according to both taxonomic group and species. The chlorophytes are shown as dark green (*D.tertiolecta*) and light green (*P.capsulatus*), the diatoms as dark brown (*P.tricornutum*) and light brown (*T.weissflogii*), the coccolithophores as black (*E.huxleyi*) and grey (*C.pelagicus*), and the cyanobacteria are shown as orange (*Synechococcus sp.*)*.* Pairwise competitive responses are shown along the x axis and full community competitive responses are shown along the y axis. All points displayed are mean values with ± 1 standard deviation (N=12). The greatest responses were always exhibited by the chlorophytes, followed by the diatoms (in batch high-nitrogen conditions) or coccolithophores (in semi-continuous lower-nitrogen conditions) and the lowest responses were always exhibited by the cyanobacteria.
